# Sources, Production, and Clinical Treatments of Milk Fat Globule Membrane for Infant Nutrition and Well-Being

**DOI:** 10.3390/nu12061607

**Published:** 2020-05-30

**Authors:** Javier Fontecha, Lauren Brink, Steven Wu, Yves Pouliot, Francesco Visioli, Rafael Jiménez-Flores

**Affiliations:** 1 Food Lipid Biomarkers and Health Group, Institute of Food Science Research (CIAL, CSIC-UAM), 28049 Madrid, Spain; 2 Department of Medical Affairs, Mead Johnson Nutrition, Evansville, IN 47721, USA; Lauren.Brink@rb.com (L.B.); Steven.Wu2@rb.com (S.W.); 3 Department of Pediatrics, Indiana University School of Medicine, Indianapolis, IN 46202, USA; 4 STELA Dairy Research Center, Institute of Nutrition and Functional Foods (INAF), Department of Food Sciences, Laval University, Québec, QC G1V 0A6, Canada; Yves.Pouliot@fsaa.ulaval.ca; 5 Department of Molecular Medicine, University of Padova, 35121 Padova, Italy; francesco.visioli@unipd.it; 6 IMDEA-Food, CEI UAM + CSIC, 28049 Madrid, Spain; 7 Food Science and Technology Department, The Ohio State University, Columbus, OH 43210, USA

**Keywords:** milk fat globule membrane, polar lipid composition, sources, production, clinical studies, infant nutrition, infant formula

## Abstract

Research on milk fat globule membrane (MFGM) is gaining traction. The interest is two-fold; on the one hand, it is a unique trilayer structure with specific secretory function. On the other hand, it is the basis for ingredients with the presence of phospho- and sphingolipids and glycoproteins, which are being used as food ingredients with valuable functionality, in particular, for use as a supplement in infant nutrition. This last application is at the center of this Review, which aims to contribute to understanding MFGM’s function in the proper development of immunity, cognition, and intestinal trophism, in addition to other potential effects such as prevention of diseases including cardiovascular disease, impaired bone turnover and inflammation, skin conditions, and infections as well as age-associated cognitive decline and muscle loss. The phospholipid composition of MFGM from bovine milk is quite like human milk and, although there are some differences due to dairy processing, these do not result in a chemical change. The MFGM ingredients, as used to improve the formulation in different clinical studies, have indeed increased the presence of phospholipids, sphingolipids, glycolipids, and glycoproteins with the resulting benefits of different outcomes (especially immune and cognitive outcomes) with no reported adverse effects. Nevertheless, the precise mechanism(s) of action of MFGM remain to be elucidated and further basic investigation is warranted.

## 1. Introduction

Breastfed infants have a lower incidence of all-cause and infection-related mortality than their bottle-fed counterparts [1]. Human milk (HM) provides the newborn with the nutrients (proteins, long-chain fatty acids, carbohydrates, vitamins, minerals, etc.) and immune protection (antibodies and oligosaccharides) needed to promote optimal growth and development. Among many other important components of HM, an increasing body of evidence shows that the milk fat globule membrane (MFGM) and its constituents support the education and development of the immune system of infants [2,3,4,5,6], which is attributed to both the presence of cholesterol and polar lipids, including phospho- and sphingolipids, and to the abundance of glycosylated and non-glycosylated proteins. Because MFGM can exert diverse positive health effects on immune and GI outcomes, brain development, gut functionality, metabolism, and cognition, as reported in human and animal studies [7,8,9], it seems appropriate to think that the addition of MFGM to infant formula (IF) could bring the product closer to approximating HM composition and function.

MFGM is a complex structure made of lipid droplet buds originating from the endoplasmic reticulum of lactocytes, forming a phospholipid monolayer vesicle that helps emulsify lipids in the aqueous cytoplasm. As the lipid droplets reach the apical cell membrane, an additional bilayer from the lactocyte plasma membrane encases the fat droplet during extrusion from the cell [10]. Therefore, MFGM is structurally composed of a triple layer of phospholipids and cholesterol with incorporated proteins and glycoproteins and it maintains the physical, chemical, and functional characteristics of the apical surface membrane of the mammary epithelial cells from which it originates.

Research on MFGM has been gaining momentum due to (1) its unique trilayer structure and composition, namely the presence of phospho- and sphingolipids and glycoproteins; (2) the search for novel supplements/nutraceuticals and bioactive ingredients to be employed in several circumstances; and (3) the opportunity to source MFGMs in large amounts at quite low prices. Indeed, there are two areas where MFGM could be conceivably exploited. The first one is infant nutrition because formula-fed infants have much lower intakes of MFGM than breastfed ones [11]. This lower MFGM intake is due to the replacement of milk fats with vegetable oils in IF. The addition of MFGM to IFs allows for the provision of nutrients that play important roles in neural and cognitive development. Examples of these nutrients include gangliosides, sialic acid, and sphingomyelin. Further, these molecules contribute to the proper development of immunity and intestinal trophism, possibly mediated by the evolving microbiota. The second area where MFGM is being positioned is the prevention of several diseases including cardiovascular disease [12], impaired bone turnover and inflammation [13], skin conditions [14], and infection by *E. coli* [15], as well as age-associated diseases such as cognitive decline and muscle loss. Stemming from the observation that the phospholipid composition of MFGM is quite similar to that of neuronal cell membranes, some investigators are proposing the use of MFGM to counteract the loss of some neuronal components such as polyunsaturated fatty acids (PUFAs), namely those of the omega-3 series [16,17,18,19].

In view of the aforementioned increasing relevance of MFGMs, this review focuses on the human (namely infant) and animal research that collectively suggests that MFGM and its components show efficacy on different aspects of human health. In particular, we focus on the risk–benefit of using ingredients enriched with MFGM or milk phospholipids in IF [20].

## 2. Sources, Production, and Treatments of Dairy-Based Ingredients Containing Milk Fat Globule Membrane (MFGM)

The potential use of MFGM to design emulsions for dairy products and IFs has been reviewed by several authors over the last decade [4,21,22,23,24,25,26,27,28]. MFGM structure and function characteristics are still not fully elucidated; however, a number of technological approaches to obtain MFGM isolates or to generate MFGM-enriched dairy ingredients have been brought to commercial scale and the use of these ingredients as supplements for IFs or other MFGM-enriched products is now feasible.

Dairy-based ingredients containing MFGM fragments including minor lipids fall in two categories, namely MFGM-enriched ingredients and phospholipid extracts [29]. MFGM-enriched ingredients are obtained by a combination of physical processes, whereas most of the phospholipid extracts are obtained by solvent extraction from MFGM-enriched fractions [30]. Phospholipid extracts do not contain MFGM fragments and instead are used in cosmetic and skin-care applications, whereas MFGM-enriched ingredients are better designed for nutritional applications.

**Figure 1:**
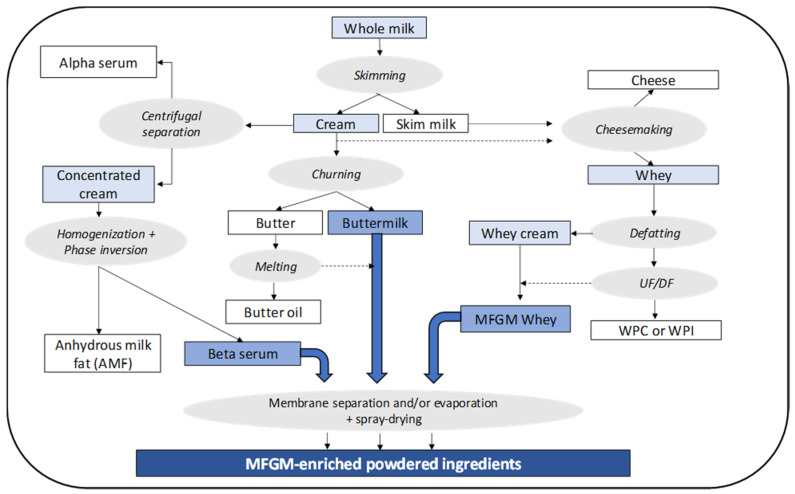
Processing alternatives to produce milk fat globule membrane (MFGM)-enriched powdered ingredients.

Destabilizing the milk fat globule natural emulsion is at the basis of most of the commercial processes to produce MFGM-enriched ingredients [31,32]. As illustrated in Figure 1, cream that is obtained by skimming whole milk constitutes the raw material for butteroil or anhydrous milk fat (AMF) and butter manufacturing. Beta serum and buttermilk, the co-products of these two dairy foods, contain most of the MFGM components, including minor lipids and MFGM proteins. Cheese whey, the co-product from cheesemaking processes, is also considered a potential source of MFGM-enriched ingredients since it contains residual fat mainly composed of milk minor lipids and MFGM proteins. This fat must be removed from the whey feed before the valorization of whey proteins as whey protein concentrates (WPC) or whey protein isolates (WPI). The phase inversion obtained by churning of a fat cream generates butter grains mainly composed of triacylglycerol (TAG) together with buttermilk, an aqueous phase having an overall composition similar to that of skim milk but also containing MFGM constituents. Buttermilk downstream processing can simply consist of evaporation and spray drying, but membrane separation processes can be applied in order to increase the protein and MFGM content of the MFGM-enriched powder.

MFGM-enriched ingredients can also be obtained by additional two methods. One method occurs during the AMF production process, after destabilization of concentrated cream in which the fat content of cream is raised from 35–45% to 75% by centrifugal separation and the cream concentrate is then fed to a homogenizer where phase inversion occurs. This process produces a >99.5% fat phase and an aqueous phase called beta serum that contains all of the MFGM components. The second method involves melting and holding butter at 60 °C for 30 min, followed by centrifugal separation leading to a >99.5% fat oil phase and an aqueous phase of buttermilk containing MFGM constituents.

As mentioned earlier, another source of MFGM is cheese whey by-products. Cheese whey typically contains 0.5–0.8% protein, 4.5–5.0% lactose, and 0.1–0.5% residual fat. The fat level is highly dependent on the (standardized) cheese milk composition and on the characteristics of the cheesemaking process. The residual fat found in cheese whey is mainly composed of minor lipids and free fatty acids, although some small droplets of TAG released from the cheese matrix can occur in whey. Downstream whey processing aims at recovering and purifying whey proteins as functional ingredients by ultrafiltration (UF) and/or microfiltration (MF) and, for this purpose, the whey feed must be defatted prior to membrane processing in order to prevent membrane fouling. Whey cream can be generated by classical centrifugal separation; however, complete defatting of whey is advised for downstream ion-exchange chromatography in the manufacture of WPI. Such defatting can be achieved using thermo-calcic aggregation that consists of the addition of CaCl_2_ and pH adjustment [33,34]. The aggregates generated by this process can be collected by a soft stream separator or by milk fat (MF) processing. In both cases the aggregated phase contains a higher proportion of MFGM constituents. Whey processing also offers the possibility to tailor MFGM-enriched ingredients with higher whey protein contents.

Several alternative methods or approaches to prepare MFGM-enriched ingredients developed at laboratory or pilot scale have been reviewed [26,28,35,36,37]. Although these methods offer some interesting features, their scale-up is often limited by technological or economic considerations. Among these alternative methods, washing the cream, i.e., diluting and re-concentrating the cream by centrifugation is still considered promising from the point of view of MFGM yield [38]; however, the performance is not well documented. Another approach is to process whey cream into whey butter and buttermilk. In the latter case, the membrane-based concentration process to derive MFGM is facilitated by the absence of casein micelles in whey buttermilk [39,40].

Some dairy ingredients manufacturers have developed proprietary processes to produce MFGM-enriched ingredients and a relatively wide array of ingredients (in terms of composition) can be found on the international market. Table 1 summarizes the overall compositional characteristics of commercial MFGM-enriched ingredients currently available and prepared from co-products of butter, anhydrous milk fat, or cheese whey. These ingredients not only differ from each other in terms of overall composition (protein, lactose, ash, total lipids, and phospholipids), but the proportion of each type of phospholipid is also different. Beta serum appears to be the ingredient offering the highest phospholipid content (≥16%) in contrast with buttermilk, which has the lowest content (1.6%). MFGM whey offers the possibility of providing up to 73% protein while still containing 7.5% phospholipids. This high protein content represents an interesting feature in the context of nutritional products such as IF or other dairy products. The detailed protein composition of MFGM-enriched ingredients is not systematically disclosed in technical data sheets; however, most the ingredients prepared from cream, beta serum, or buttermilk contain measurable amounts of gangliosides, MFGM proteins, cholesterol, sialic acid, IgG, and lactoferrin [41,42,43,44,45].

The ingredients listed in Table 1 describe the major differences in compositional characteristics according to the source of MFGM. It must be emphasized that each category of ingredient can be tailor-made accordingly to meet the end-user specifications. The ingredients manufacturer can adjust the composition of the final ingredients by process control (centrifugation speed and temperature, UF/MF concentration, etc.). Several studies have shown that the polar lipid profile of MFGM-enriched streams from buttermilk, butter serum, or MFGM whey can be modified by adjusting technological conditions [37,47,48,49,50,51,52]. Finally, the approach of blending different feeds (for example, beta serum, WPI, other milk solids) can also lead to tailored MFGM-enriched ingredients for specific applications, such as IFs.

## 3. Biological Effects of MFGM

The composition, health attributes, and biological activity of commercial ingredients containing MFGM, milk phospholipids, or MFGM isolated in the laboratory have been extensively reviewed [2,5,22,25,53,54,55,56,57,58,59]. Given the complexity of the composition of MFGM, here we present in a differentiated way the available information on health benefits of (a) proteins and (b) lipids, in particular phospholipids.

### 3.1. Proteins of the MFGM

The main proteins associated with the MFGM from its genesis in lactation have been identified and all of them have been sequenced by identification of their genetic code. Table 2 summarizes such proteins and their molecular weights.

There are several aspects that must be underscored: (1) All proteins are glycosylated except ADPH and FABP, which are also of smaller molecular weight, and (2) in general, there are other important proteins associated with MFGM. Butyrophilin and XDH/XO constitute about 40% and 12% of total MFGM proteins, respectively, and mucins are highly sialylated proteins, with about half of their weight made up of carbohydrates. These proteins are in milk and, whether by methodology of isolation or by natural affinity, they are generally present in any laboratory or industry concentration or isolation of MFGM proteins. Whey proteins, especially beta-lactoglobulin, lactoferrin, and immunoglobulins, are commonly associated with MFGM [60,61]. Therefore, it is important to consider the method of isolation and a detailed composition of the ingredient used to study the properties of MFGM (as described in the previous section).

Table 3 summarizes the studies that support some of the health benefits of the MFGM proteins (as individually studied related to their biological activity).

### 3.2. Lipids of the MFGM

To date, there are still many MFGM lipid components in both human and bovine milk that continue to be further investigated in terms of their structure, function, role, and biological significance to human health. Examples of such components are glycerophospholipids (as phosphatidylcholine (PC), phosphatidylethanolamine (PE), phosphatidylinositol (PI), and phosphatidylserine (PS), and sphingolipids (especially sphingomyelin, SM)), glycolipids (cerebrosides and gangliosides), cholesterol, and other minor components [86]. The MFGM polar lipids (phospho- and sphingolipids) account for 60–70% of total milk polar lipids and their concentration (about 20 mg/100 mL) is comparable between human milk fat and bovine milk fat [87,88,89,90]. Of the major lipids present in MFGM, SM is perhaps best understood as contributing to neurodevelopment and thus is of particular interest for MFGM supplementation in infant formula milk. The range of mean SM concentrations in HM has been reported from 24.5–174 mg/L [91,92,93,94,95,96]. The phospholipids present in IF are based on vegetable fat and are generally provided by lecithin derived from either sunflower seeds or soybeans [97]; however, SM and PS cannot be sourced via plant-based fat blends and must be sourced from bovine milk.

Among the structural functions of MFGM, its ability to stabilize the MFG as an emulsion that facilitates its digestion, absorption, and metabolism stands out as a novel property since it facilitates bioaccessibility and bioavailability of naturally occurring bioactive components in MFGM [18,98]. Milk polar lipids can be found both in MFGM (which accounts for the largest quantity) and in nanovesicles that are secreted into milk by the mammary gland cells and are implicated in cell-to-cell communication by means of their functionally active cargo (such as mRNA, miRNA, and different proteins) [2].

While it is very difficult to find studies on MFGM that completely discriminate its components, many investigations on the topic focus on the phospholipids [2,99,100,101,102]. One factor unifies these studies—the conclusion that the health benefits of MFGM lipids build the grounds for their inclusion in functional foods.

### 3.3. Preclinical Evidence on MFGM Components and Ingredients

As we consider the efficacy of MFGM and its biological activity, it is important to understand the characteristics of the ingredients used for experimentation. As discussed above (and in Table 1), MFGM ingredients can derive from different starting materials. MFGM ingredients are largely similar to more commonly used dairy ingredients (buttermilk, WPC), with the main difference being that they have a much higher concentration of phospholipids and proteins originating from MFGM. In part due to its heterogenous composition, MFGM indeed has multiple mechanisms of action by which it improves human health. The predominate mechanisms that have been studied in infant health are: anti-pathogenic (both through decoy or direct bactericidal activity); modulation of the intestine, mucosal immune system, and gut microbiota; and neurodevelopment. A thorough description of these mechanisms has been recently reviewed [103]. A brief description of each follows and within Table 4 descriptions of preclinical studies on MFGM ingredients can be seen.

- Anti-pathogenic effects

Anti-pathogenic effects of MFGM may derive from either direct bactericidal activity or interference with pathogen adhesion to the intestinal epithelium (decoy effect), thus preventing these pathogens from either access into the body or the initiation of physiologic cascades leading to adverse effects such as infection-induced diarrhea and inflammation. There is a large body of literature investigating how MFGM and its components may reduce the burden of common infectious diseases including: Escherichia coli [15,123,124,125,126,127,128,129], Salmonella enteritidis [112,124,130,131], Listeria monocytogenes [112], and rotavirus [132,133]. These studies provide evidence that direct binding to and inhibition of GI pathogens by MFGM can occur within the gut lumen and are consistent with the absence of evidence for MFGM-related toxicity.

- Intestinal epithelial, mucosal immune system, and gut microbiota

In addition to direct interactions with GI pathogens, MFGM and its components have also been studied for their effects on gut immune, microbial, and epithelial development. The potential contributions to the infant immune system by MFGM and its individual components, such as lactadherin and gangliosides, have been reviewed [5]. The most commonly reported immune modulatory effects consist of shifts in balance favoring anti-inflammatory over inflammatory cytokines [134,135,136], providing protection from inflammatory damages within the intestine [114,115,116,117,137], and modulating the gut microbiome [109,118,119,120,121,122,138,139]. The role of MFGM in modulating the microbiome is still currently an ongoing discussion, as many of the studies cited have multiple nutritional interventions (other prebiotics or different fat sources). Nonetheless, these studies support the addition of MFGM to IF in order to support the gut and immune development of infants.

- Neurodevelopment

Building upon the observation that there has historically been a gap in some cognitive outcomes between breastfed and formula-fed infants, a variety of preclinical studies have provided evidence for the mechanisms by which MFGM components, specifically polar lipids and sialic acid, and ingredients support the development and proper functioning of the central nervous system [140,141,142,143,144,145,146,147,148]. All the publications support the addition of MFGM to IF in order to provide long-lasting cognitive benefits and narrow the gap between formula fed and breastfed infants.

In summary, the literature on MFGM-enriched ingredients has been shown in multiple preclinical model systems to demonstrate consistent support for normal brain, immune, and gut development function (Table 4.).

## 4. Comments on Clinical Studies

**Figure 2:**
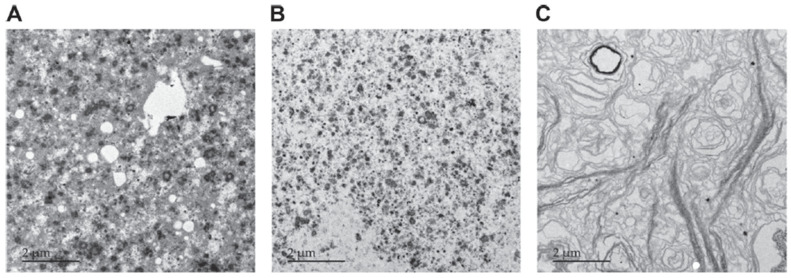
Electron microscope image of three different commercial ingredients containing MFGM. Each sample varies in composition and concentration of phospholipids and in structure. However, all of them have been used successfully in food products all over the world (adapted from ref [2]).

As we reported previously [2], the structure and composition of different ingredients containing MFGM vary widely, as shown in Figure 2.

In Table 5 we provide a summary of all the clinical trials on MFGM and its components for infants and young children. However, given the variety in structure and composition of MFGM ingredients, we consider it important to focus in detail on some of the reported clinical studies on cognitive development and other measures for a single ingredient defined as WPC enriched with MFGM. Several double-blind controlled trials have investigated safety, growth, neurodevelopment, and health/adverse event outcomes in infants and young children consuming the WPC-MFGM ingredient. In line with the findings from animal studies, the studies we review below have demonstrated that such consumption is safe, well tolerated, and lacks evidence of adverse effects to growth, cognition, behavior, gastrointestinal health, or immunity. In fact, where differences have been found between MFGM-fed groups and control groups, they have supported the potentially beneficial outcomes of consuming supplemental MFGM.

In 2011, Zavaleta et al. reported the effects of an MFGM-enriched complementary food on health outcomes in older infants, enrolling 499 primarily breastfed term infants between 6 to 11 months of age [7]. Results showed no differences between groups in growth or serum markers (anemia, ferritin, zinc, or folate). Furthermore, the group receiving MFGM had a significantly lower prevalence of diarrhea vs. the control group (3.8% vs. 4.4%), as well as a significant reduction (46%) in episodes of bloody diarrhea.

Several follow-up publications have been made in response to the Zavaleta study. A recent follow-up publication on this trial demonstrated specific metabolome and immune-related outcomes in infants who consumed MFGM. These infants (6–11 months of age) displayed a decreased Th1 response that was attributed to lowered serum cytokine IL-2. Thus, this study further corroborates the immune outcomes found in the trial as the Th1 immune system is activated during infection [150]. In another study, Gurnida et al. (2012) studied the effects of formula supplemented with a ganglioside-enhanced, MFGM-derived, complex milk lipid in term infants [151]. Healthy infants (2–8 weeks) were fed until 6 months of age with either standard control IF (*n* = 30) or a supplemented IF (*n* = 29) with added MFGM-derived lipids to increase total ganglioside concentration from 6 to 9 mg/100 g. The measured level of gangliosides in the supplemented formula fell within the range reported for HM. A breastfed reference group (*n* = 32) was also included. There were no differences in any growth parameter between the two formula groups. There were no differences in reported morbidities between the two formula groups, including symptoms such as fever and cough, diarrhea, allergy, vomiting, or colic. There were also no adverse effects on developmental milestones; conversely, the test formula group had increased scores on three domains of the Griffiths Mental Development Scale at 6 months compared to the control group and did not significantly differ from the breastfed reference group.

A study with slightly older children, 2.5 to 6 years old was reported by Veerman-Wauters et al. [165]. In this trial, the children consumed a fortified milk beverage (in addition to usual diet) for a period of four months. The control group beverage (*n* = 97) contained 60 mg/day of endogenous phospholipid, while the test group beverage (*n* = 85) contained a total of 500 mg/day of phospholipid due to the addition of MFGM concentrate. Both beverages were reported as well-tolerated with no differences in symptoms or health outcomes between groups. The questionnaire for evaluating safety and potential problems included rash, vomiting, diarrhea, constipation, cough, varicella, otitis, pharyngitis, doctor visits, days of school absence, or use of medication. Statistically significant reductions were found only for days with fever and number of short febrile episodes (<3 days), both in favor of the MFGM-supplemented group. The primary outcome, the validated Achenbach behavioral-emotional assessment questionnaire (ASEBA), was completed by parents and teachers; the only significant difference found was lower behavioral problem scores, as determined by parent assessment, in favor of the MFGM group.

There is also a study that focused on the safety and tolerance of MFGM when added to IF [159]. This trial investigated growth and tolerance in healthy term infants receiving standard control formula (*n* = 57) or formula enriched with a protein-rich MFGM fraction (MFGM ingredient, *n* = 72) or a lipid-predominant MFGM fraction (MFGM-L, *n* = 70) up to 4 months of age [159]. Growth and formula tolerance did not differ between groups. Overall incidence of adverse health events or serious adverse health events did not differ statistically between groups, except for post hoc testing reporting a higher rate of eczema in the MFGM ingredient-fed group (13.9% vs. 3.5% in control and 1.4% in MFGM-L). The authors recognized limitations to the interpretation of this finding, concluding: “Although...adequately powered to evaluate non-inferiority...with respect to weight gain, limitations of the study for investigating other outcomes include its short duration and relatively small sample size, as well as the unequal allocation of subjects among groups, which may have introduced some degree of bias between the control and the experimental groups”. It is also observed that for several other reported events, incidence was lower in the MFGM-ingredient group including otitis media (2.1-fold lower) and oral candidiasis (6-fold lower), though these did not reach statistical significance.

Perhaps the most important and highly cited papers on this topic are the two published by Timby et al. in 2014 and 2015. These studies aimed to determine the effects of supplementation with MFGM on growth, health, and cognitive outcomes [154,156]. Term infants (<2 months old) were assigned to consume either a standard formula (*n* = 80) or an MFGM-supplemented formula (*n* = 80) until 6 months of age. The investigational formula contained MFGM-10 at a dose of 6 g/L. A breastfed reference group (*n* = 72) was also included. Results demonstrated that growth, tolerance, and safety were equivalent between formula groups. Comparison of adverse events, disease symptoms, and medication use demonstrated that the incidence of acute otitis media during the intervention was significantly decreased in the MFGM group compared to control (1% vs. 9%), and not significantly different from the breastfed reference group (0%). No other differences in other infections or health outcomes were seen; in particular there was no significant difference in incidence of eczema or any skin rash (17% in the MFGM group vs. 26% in control). The only difference in medication use was lower antipyretic drug use in the MFGM group. Serum anti-pneumococcal IgG titers were measured as a secondary endpoint. Of 10 measured serotypes, the MFGM group had lower concentrations for types 1, 5, and 14 compared with the control group, though the authors note that for all serotypes pneumococcal titers were also lower in the breastfed reference group than in the control formula. Furthermore, it can be observed that for 8 of 10 serotypes, mean antibody levels in the MFGM group are closer than the control group to breastfed infant levels. Cognitive assessment at 12 months of age with the Bayley-III Scales showed significantly higher mean cognitive domain scores in the MFGM-fed group vs. the control group, with no significant differences in the four other developmental domains, demonstrating that there was no evidence for adverse effects to either health or neurodevelopmental outcomes. Investigations into the cardiometabolic profile [155], oral microbiome [157], the fecal microbiome and metabolome [166], serum metabolome [166], and serum, plasma, and erythrocyte lipidome [167] have additionally been described.

The latest papers that have an in-depth study and large number of subjects are those from Li, X. et al. [9] and Li, F. et al. [163]. The first study evaluated the effects on growth and infectious outcomes of IF supplemented with either MFGM or a probiotic (*L. paracasei* ssp. *paracasei* strain F19) [9]. A total of 600 infants were randomized to consume either a standard control formula, formula supplemented with MFGM-10, or formula with probiotic (*n* = 200 per group) for 4 months. A breastfed reference group was also recruited (*n* = 200), and all infants were followed until one year of age. Both experimental formulas were well tolerated and supported normal growth. Adverse event rates were highest in the control formula group. Overall, during the intervention, the MFGM-formula group did not have significantly more diarrhea, fever, days with fever, clinic visits, or upper respiratory infection (URI) episodes than the other formula groups or the breastfed infants. In contrast, the control formula group (without MFGM) had significantly more fever episodes and days with fever than the breastfed reference group.

A separate study was designed to evaluate the growth, health, and cognitive effects of formula containing both MFGM and lactoferrin (Lf) [163]. Healthy term infants were fed through 12 months with either routine control formula (*n* = 208) or a formula containing MFGM-10 at 5 g/L + lactoferrin 0.6 g/L (*n* = 198). Unlike prior studies, children were followed to 18 months of age after finishing the study formula consumption at 12 months. Results showed no significant differences between groups in growth, tolerance, stool characteristics, or antibiotic use; the MFGM+Lf group had significantly lower incidences of GI and respiratory adverse events (including diarrhea, URI, and cough) compared to the control group. Again, there were no differences in reported incidence of skin events or eczema, notably in a study with a much larger population size and longer follow-up period than the Billeaud 2014 study. Finally, cognitive outcome measures showed that the MFGM+Lf formula group had higher cognitive, language, and motor Bayley-III scores at 12 months, longer sustained attention at 12 months, and higher scores on some elements of language performance at 18 months compared to the control formula group.

In summary, the body of clinical studies to date that test the dietary effects of an MFGM-enriched diet in infants and young children have consistently shown that the diets are well-tolerated and support normal growth, health, and neurodevelopmental outcomes. The studies summarized here included over 1000 children assigned to MFGM supplementation. Seven of these studies [7,9,155,156,159,161,165] initiated feeding with MFGM-enriched formula at <2 months of age, and five [7,157,159,167] used the same source of MFGM ingredient. Though the majority of studies were designed to follow health and safety outcomes to endpoints of 12 or 18 months, the longest-term follow-ups to date from MFGM feeding in infancy now extend to 6 and 13 years of age [11]. Overall, this collective clinical evidence strongly supports the safety of MFGM supplementation in the infant and young child populations and has not highlighted specific concerns for adverse pediatric outcomes. Indeed, the data is more consistent with beneficial effects of consuming supplemental MFGM as compared to formulas with only inherent amounts of MFGM.

## 5. MFGM-Enriched Dairy Ingredients: Critical Unit Operations and Safety Concerns

All commercial MFGM-enriched dairy ingredients are manufactured from regular dairy products (butter, cream, or cheese) or from their by-products. The processing approach for the manufacture of MFGM-enriched dairy ingredients involves two categories of unit operations, namely: (i) Physical processes: churning, pressure-driven membrane separation (UF or MF), centrifugal separation (skimming or concentrating cream), and (ii) Operations involving heating: high temperature/short time (HTST) pasteurization, sterilization, evaporative concentration, spray-drying. Other operations involving the use of chemical additives such as CaCl_2_ for defatting cheese whey can also be involved, but this substance does not represent safety concerns since it is already used in most of the cheese manufacture processes that generate cheese whey.

All dairy ingredient manufacturers must implement procedures such as the Hazard Analysis Critical Control Point (HACCP) procedures to avoid any product microbial or chemical contamination of the ingredients. Heat-treatments such as pasteurization (72–75 °C/16 s) and other HTST heat treatments (for example, 115–135 °C/20 s to 2 min.) are the most important critical control points with regards to product safety in ingredients manufacture [29]. Typical MFGM-enriched dairy ingredients must meet microbial specifications in terms of standard plate count (<10,000/g), *Enterobacteriaceae* (neg/g), *Salmonella* (neg/750 g), *Bacillus cereus* (<100/g), and yeast and mold (<200/g). However, additional contaminant microorganisms (such as *Cronobacter*) may be included as specification from the perspective of using these ingredients in IFs.

## 6. Regulatory and Safety Aspects of Infant Formula (IF) Added Ingredients

In recent years, IF manufacturers have added ingredients such as omega-3 PUFAs and probiotics to their products. These innovations have created new regulatory challenges. Some algorithms are being proposed and the fast pace at which new bioactive compounds are being introduced to the market indeed calls for action [168].

According to the FDA, approximately one million infants in the United States are fed IF from birth and by three months of age about 2.7 million infants rely on IF as part of their nutrition. Of note, the agency does not approve IFs before they can be marketed. In June 2014, the FDA issued the final rule “Current Good Manufacturing Practices, Quality Control Procedures, Quality Factors, Notification Requirements, and Records and Reports for Infant Formula,” which requires manufacturers of IF to use current good manufacturing practices specifically designed for IF. In addition, producers must test for the presence of noxious microbes such as *Salmonella* and/or *Cronobacter*, demonstrate that their IFs support physical growth and that the protein component is of adequate biological quality (this quality is based on Regulations under Sections 201(s), 201(z), and 412 of the Federal Food, Drug and Cosmetic Act), and test IFs for nutrient content before they put it on the market and again at the end of the product’s shelf life. The new formula must be notified to the FDA at least 90 days before being commercialized.

In Europe, the Commission mandates the European Food Safety Authority (EFSA) to assess the safety of xeno-ingredients added to foods, granting the Novel Food status to constituents of proven safety at habitual levels of intake [169,170]. The legal framework is that of Regulation (EU) 2015/2283 (25 November 2015) of the European Parliament and of the Council on Novel Foods and the Commission Implementing Regulation (EU) 2017/2469. The overarching goal is that of an EFSA project on a customer-oriented approach for regulated products aiming at supporting applicants and other stakeholders during the whole life cycle of the applications for regulated products. The aforementioned document includes a completeness checklist that the applicant first and the EFSA later use to verify the completeness of the data for risk assessment in the technical dossier [170]. Four summary tables are also included for applicants to summarize the results of the scientific studies provided in the technical dossier. As regards IF, they are covered by Commission Directive 2006/141/EC and its subsequent amendments. As of 2021, IF will be regulated by Commission Delegated Regulation (EU) 2016/127, which is built on the observation that “Infant formula is the only processed foodstuff which wholly satisfies the nutritional requirements of infants during the first months of life until the introduction of appropriate complementary feeding.” This regulation specifically mentions omega-3 PUFAs and their minimum and maximum amounts.

In Australia, the Food Standard Australia New Zealand (FSANZ) authority regulates novel foods in a manner similar to the EFSA one. Of note, in Australia and New Zealand, novel foods and novel food ingredients are regulated under Standard 1.5.1-Novel Foods in the Food Standards Code. A novel food cannot be sold as food or used as a food ingredient unless it is listed in that Standard, which is continuously amended.

As far as MFGMs are concerned, to the best of our knowledge, there is only one published, small sample size, non-inferiority trial [159] that evaluated a lipid-rich MFGM concentrate (MFGM-L) and a protein-rich MFGM concentrate (MFGM-P) versus standard formula. In post-hoc analysis, the authors reported a higher (13.9%) rate of eczema in the MFGM-P group compared to the control (3.5%) and MFGM-L (1.4%) groups. The authors commented that their findings should be interpreted with caution and, as noted previously, no increased risk of eczema or rash were demonstrated in two subsequent larger and longer-term feeding studies using the same source (MFGM-10) at equivalent levels [8,9]. A possible interpretation for this discrepancy might be the current lack of a systematic eczema scoring system, which is being developed [171]. In other words, eczema is usually assessed based on parental reports, daily records, and physician examinations, which are not standardized. In addition, the lipid component of MFGMs is, conceivably, devoid of side effects even though some authors suggest an adjuvant effect based on uncertain biological mechanisms [172]. Conversely, the protein component of MFGMs could be implicated in some allergic reaction, although there are no clear indications of such an effect.

In summary, while the existing clinical data generally support the safety and, in several regards, the benefits of supplementing MFGM in infants and young children, the current lack of harmonization between various regulatory frameworks as well as the relatively limited number of human trials targeted at both effectiveness and safety of MFGM impede us from drawing firm conclusions. Additional well-designed, large-scale future trials will be necessary in order to fill the gaps in knowledge about MFGM and to provide more definitive evidence for safety.

## 7. Future Developments

MFGM-enriched ingredients are now available for use in IFs. Further research should address the processing of these MFGM enriched ingredients, including:

- Better characterization of minor MFGM proteins and their content along with their variability, as influenced by the processing conditions of the ingredients.
- Demonstrate and ensure that MFGM systems deliver bioactivities to the infants in a reproducible manner (process control vs. variability of raw material).
- Develop sustainable processes as alternatives to solvent extraction to increase the phospholipid or minor lipid content from MFGM-enriched ingredients.

In terms of clinical investigations, though evidence to date has been promising, there remains a need for further randomized trials with well-defined neurocognitive and safety endpoints in order to better understand the effects of supplemental MFGM in infant feeding. Given the essential role of appropriate infant nutrition to health and development throughout childhood and beyond, such studies merit attention from researchers, public health bodies, and funding agencies. Furthermore, when considering the variety of other potentially bioactive molecules present in human milk including oligosaccharides, lutein, and other carotenoids, it is possible to consider that these may work additively or synergistically with MFGM to provide benefits [18].

## 8. Conclusions

In this Review we arrive at the following conclusions: the source for MFGM in all the studies presented has its origin in bovine milk, and although there are some differences in processing, the final composition of MFGM ingredients is within a limited range where the phospholipids present in the ingredients are very similar to the composition of these in milk. The literature also shows that the processing involved in concentrating the different ingredients does not result in a chemical change—these processes result in concentration of the components originally found in native MFGM of bovine milk. The ingredients, as used to improve the formulations used in all the different studies, have indeed increased the presence of phospholipids, sphingolipids, glycolipids, and glycoproteins, with the resulting benefits of different outcomes (especially immune and cognitive) sought in the various studies. However, although there are remarkable and measurable benefits, the precise mechanism that leads to these benefits remains to be elucidated. One aspect that seems to be evident is that there are no reported adverse effects that would increase the health risk of using these ingredients.

## Figures and Tables

**Table 1:** Commercially available MFGM-enriched dairy ingredients.

	MFGM-Enriched Ingredients		
(g/100 g Product)	Buttermilk	Beta Serum	Whey
*Source/process*	*Butter*	*Anhydrous milk fat*	*Cheese whey*
Protein (N × 6.38)	≥30	>52	73
Lactose	±50	≤10	≤3
Ash	≤9	≤6	≤3
Total lipids	5–13	3–27	12–26
Total phospholipids (PL) (g/100g fat)	1.6–22	≥14	5–16
Phospholipids (% of total PL)			
- Phosphatidyl ethanolamine (PE)	35–43	22–29	19–41
- Phosphatidyl choline (PC)	19–32	27–47	19–25
- Phosphatidyl serine (PS)	8–18	1.2–23	8–12
- Phosphatidyl inositol (PI)	4–9	1–8	3.6–7
- Sphingomyelin (SM)	11–19	14–27	16–24
- Others	-	<4	<7
Moisture	≤4%	≤5%	≤6%
pH	6.0–6.5	6.2–6.8	6.4

Average lipid composition based on refs [29,35,37,46].

**Table 2:** Proteins native to the MFGM.

Protein Common Name	Molecular Weight
BRCA1 and BRCA2	210
Mucin I (MUC1)	160–200
Xanthine oxidase (XO)	146–155
PAS III	94–100
CD36	76–78
Butyrophilin (BTN)	66–67
Adipophilin (ADPH)	52
PAS 6/7 (lactadherin)	47–59
Proteose peptone 3	18–34
FABP	13–15

BRCA: Breast Cancer proteins; PAS: Protein domain; FABP: fatty acid-binding protein.

**Table 3:** Biological studies of MFGM proteins (Adapted from ref [58]).

Component	Health Aspects	References
Studies in vitro and in vivo		
Mucin I (Muc1)	Antiadhesive effect	[62]
	Protective effect against rotavirus infection	[63,64]
	Effects on digestion	[65]
Xanthine dehydrogenase/	Antimicrobial agent	[66]
oxidase (XDH/XO)	Source of reactive oxygen species (ROS)/anti-inflammatory properties	[67]
Butyrophilin (BTN)	Suppression of multiple sclerosis	[68]
	Development of experimental autoimmune encephalomyelitis	[69]
	Influence on pathogenesis of autistic behavior	[70]
	Regulation of immunity	[71]
Periodic acid Schiff 6/7 (PAS 6/7) (lactadherin)	Protection from viral infections in the gut	[63,72]
	Epithelialization, cell polarization, cell movement and rearrangement, neurite outgrowth, synaptic activity in the central nervous system	[73]
Fatty acid binding	Breast cancer cells lines inhibition	[74,75,76]
Protein (FABP)	Association with breast cancer; indicator	[77,78,79,80]
Cluster of differentiation (CD36)	Anticancer properties by interacting with FABP	[75]
	Anti-inflammatory properties	[54]
Breast cancer susceptibility proteins (BRCA1 and BRCA2)	Breast cancer DNA repair process inhibition	[81,82]
MFGM proteins (complex)	Prevention of diarrhea and improvement of anemia	[7]
	Retroviral infection prevention	[83]
	Lipid digestion and cholesterol absorption reduction	[55]
Proteomic studies of MFGM in their complexity	Association and correlation studies with health or nutrition	[84]
	Comparative study of human and cow proteins	[85]

**Table 4:** In vivo evidence on MFGM ingredients for brain, intestine/immune, and microbiome development (adapted from ref [103]).

Ingredient Description	Model	Design	Primary Finding	Ref
**MFGM and brain development**				
Complex milk lipid (CML)	Young rats	Oral supplementation via gavage from PD10–80	Improved Novel Object and Morris Water Maze performance	[104]
CML-Beta serum concentrate (BSC)	Young rats	Provided orally from PD10–60 as a gelatin	Reduced latency in Morris Water Maze test; increased expression of striatal dopamine terminals and hippocampal glutamate receptors	[105]
Whey-derived MFGM (MFGM-10 Lacprodan ^®^)	Rat pups	Oral supplementation MFGM ingredient at 500 mg/kg BW until PND21	Improved neurodevelopment (increased gene expression of BDNF and glutamate-receptor) and improved behavior test performance	[106]
Whey-derived MFGM (MFGM-10 Lacprodan ^®^)	Rat pups	Oral supplementation with WPC, MFGM ingredient, phospholipid concentrate (PL-20), and sialic acid	Increased hippocampal expression corresponding to improved behavior performance in adulthood	[107]
Whey-derived MFGM (MFGM-10 Lacprodan ^®^)	Rat pups	MFGM via cannulas inserted into the stomach	Brain metabolite differences along with improved reflexes (ear and eyelid twitch, negative geotaxis, and cliff avoidance)	[108]
Whey-derived MFGM (MFGM-10 Lacprodan ^®^)	Rat pups	MFGM in combination with prebiotics and subjected to stress via maternal separation	Ameliorated stress-induced visceral hypersensitivity and improved gut-brain axis response to stress	[109]
Whey-derived MFGM (MFGM-10 Lacprodan ^®^) in a formula	Piglets	Mixture of lactoferrin, MFGM ingredient, and polydextrose galacto-oligosaccharides (PDX/GOS) for 30 days	T microvascular changes in the brain related to grey matter concentration and diffusivity within the internal capsule	[110]
Whey-derived MFGM (MFGM-10 Lacprodan ^®^)	Piglets	Milk replacer with 0, 2.5, or 5 g/L of MFGM *ad libitum* access	Higher serum cholesterol and HDL in MFGM-2.5 g/L; No differences in brain cholesterol or changes in brain macro/micro-structure	[111]
**MFGM and immune/intestinal development**				
Sweet buttermilk powder MFGM	Rats	Buttermilk powder enriched in food pre- and during infection	Reduced colonization and translocation of the pathogenic bacteria	[112]
Cream-derived MFGM	Rats	MFGM incorporated into diet for a period of 12 weeks; colon carcinogenesis rat model	Provided resistance to gut insult through significantly less aberrant crypt foci	[113]
Cream-derived MFGM	Mice	MFGM into diet for 5 weeks and injected with LPS	More resilient to intestinal inflammation, lower levels of inflammatory cytokines, and less intestinal permeability	[114]
Whey-derived MFGM (MFGM-10 Lacprodan ^®^)	Neonatal mouse	MFGM ingredient daily during suckling period and treated with a lipopolysaccharides challenge on postnatal day 21	Less inflammation (lower inflammatory cytokines, GI hist score, higher expression of GJ proteins)	[115]
Whey-derived MFGM (MFGM-10 Lacprodan ^®^)	Rats	MFGM ingredient from day 2–14 post operation 75–85% small bowel resection	Lower expression of inflammatory cytokines and NLRP3 inflammasome	[116]
Bovine MFGM with unspecified starting material	Rats	MFGM supplemented with two concentrations in a model of Necrotizing enterocolitis (NEC)	Higher concentration group (12 g/L) exhibited reduced intestinal injury (lower NEC score, lower inflammatory cytokines, and improved survival rates)	[117]
Whey-derived MFGM (MFGM-10 Lacproda ^®^) in a formula	Neonatal piglet	Mixture of lactoferrin, MFGM ingredient, and polydextrose galacto- oligosaccharides for 30 days	Improved GI development (increased enzyme activity and morphology) and lower pathogenic bacteria in the colon	[118]
**MFGM and the establishment of the microbiota**				
Whey-derived MFGM (MFGM-10 Lacprodan^®^) in a formula	Neonatal rat	Mixture of MFGM, lactoferrin, and prebiotics in a stress model	Improvements in sleep and protection against growth of *Lactobacillus rhamnosus*	[119]
Whey-derived MFGM (MFGM-10 Lacprodan ^®^)	Rat pups	MFGM in combination with prebiotics in a maternal separation stress model	No differences	[109]
Whey-derived MFGM (MFGM-10 Lacprodan^®^)	Rat pups	Formula with MFGM via cannulas inserted into the stomach’ challenged with *C. difficile* toxin	MFGM protective against *C. difficile* toxin damage	[120]
Whey-derived phospholipid concentrate (PL-20 Lacprodan^®^)	Mice	PL-20 was compared with soy lecithin for use as an emulsifier on human microbiota-colonized germ-free mice	PL showed significantly higher relative abundance of multiple bacteria and concentrations of short chain fatty acids	[121]
Whey-derived MFGM (MFGM-10 Lacprodan^®^) in a formula	Neonatal piglet	Mixture of MFGM, lactoferrin, and polydextrose galacto-oligosaccharides for 30 days	Increased abundance of *Clostridium IV, Parabacteroides, Lutispora,* and *Sutterella,* which was associated with increased total body weight	[118]
MFGM fragments from undescribed source	Neonatal piglet	Formula with MFGM fragments compared with milk fat and with vegetable oils	MFGM had increased *Proteobacteria* and *Bacteroidetes* and decreased *Firmicutes*; changes in mucosal immunity were also observed	[122]

**Table 5:** Summary of clinical research studies on MFGM and its component for infants and young children. * MFGM is classified into various types depending on its source or the bioactive components: ganglioside enriched (GNGL), phospholipids, whey protein concentrate (WPC), complex milk lipids (CML), and sphingomyelin (SM).

MFGM Type *	Methods	Results	Safety	Study
GNGL	Preterm infants (32–36 weeks) fed IF with added gangliosides (GMF, *n* = 20) (1.43 mg/100 kcal) compared to (–) gangliosides (MF, *n* = 20) for 4 weeks	Reduced levels of *Escherichia coli* in the feces of GMF compared to MF at postnatal day 7; higher fecal counts of *Bifidobacteria* at postnatal day 30 in GMF	None: No adverse events or growth differences were reported.	[139]
PL from egg	IF provided to preterm infants +/– added PLs (+*n* = 34) (–*n* = 85) in the hospital; development of NEC was observed	Infants fed PL formula developed less NEC stage II and III with similar rates of bronchopulmonary dysplasia, septicemia, and retinopathy of prematurity.	None: No difference in weight gain or formula consumption was observed.	[149]
WPC	MFGM-10 enriched formula fed daily for 6 months to infants aged 6–11 months (5.9 g/day) (*n* = 253); skim milk control (*n* = 246)	Lower prevalence of diarrhea (3.8% MFGM vs. 4.4% skim); 46% reduction of episodes of bloody diarrhea	None: No difference in growth or serum markers (ferritin, zinc, or folate) were observed.	[7,150]
GNGL CML	IF with GNGL enriched CML (*n* = 29) fed to healthy infants (2–8 weeks old) until six months old; compared to non-supplemented formula (*n* = 30) and BF reference group (*n* = 32)	Test group had increased behavioral test scores on the Griffiths Mental Development Scale at 6 months.	None: No differences in growth or tolerance between the two formula groups.	[151]
SM	IF with SM (*n* = 12) or control (*n* = 13) fed for 8 weeks to low birth weight infants (<1500 g); evaluations up to 18 months	Increased SM in total PLs and improved scores on Behavior Rating Scale of the BSID-II, the Fagan test scores, the latency of visual evoked potentials, and sustained attention test scores at 18 months	None: No adverse events or growth differences were reported.	[152]
GNGL CML	Supplement 2 g CML + 3 g whole-milk power or control 5 g whole-milk powder for 12 weeks to infants age 8–24 months	Lower duration of rotavirus diarrhea and prevalence of major illness in the CML group was observed.	None: There were no difference in adverse events or growth between groups.	[153]
WPC	IF supplemented with MFGM-10 at 6g/L (*n* = 80), standard formula (*n* = 80), and BF reference group (BF) (*n* = 72) fed to term infants <2 months old until 6 months of age	MFGM-fed group showed significantly higher mean cognitive domain scores vs. the control group at 12 months of age; overall phenotypes observed to be more similar to BF group	None: No difference in eczema or any skin rash reported. MFGM formula supported growth and was well tolerated. Lower incidence of otitis media and antipyretic drug use reported. Serum antibody levels were more similar to BF group.	[154,155,156,157,158]
WPC	IF supplemented with MFGM-10 (*n* = 72), MFGM-L “lipid rich” (*n* = 70), or SF control (*n* = 57) fed to infants ≤14 days old at enrollment and provided until 4 months of age.	No significant differences were found for plasma PLs, cardiolipin, cholesterol ester, IGF-1, or leptin. No differences were observed in polio or HiB antibodies, with the exception of lower mean polio virus type 1 IgG level in the MFGM-10 in one group. No differences were found between groups in fecal immune markers, including alpha-1-antitrypsin, secretory IgA, and calprotectin.	No difference in adverse events or growth and tolerance. However, post-hoc analysis reported rates of eczema were higher in MFGM-10^®^ group (13.9% vs. 3.5% in SF). The short duration and relatively small sample size, as well as the unequal allocation of subjects among groups, which may have introduced some degree of bias between groups.	[159]
Cream MFGM	IF supplemented with MFGM concentrate compared to control formula and BF reference from <2 months of age and 2 months of consumption.	Concentration of serum LDL and cholesterol in MFGM-fed infants was comparable to the BF reference group.	None: No adverse events were reported.	[160]
CML MFMG	IF with MFGM (*n* = 226) fed to full term infants <14 days old up to 12 months and compared to BF reference group (*n* = 206).	Growth and dropout rate had similar scores between FF infants and BF reference at birth and 6 months. Behavioral tests, serum GNGL, and gut microbiota were measured.	None: No differences in weight between groups were reported.	[161]
MFGM within goat milk fat	IF containing either goat milk fat/plant oil mixture (GIF) or control formula with only plant oil fat provided to healthy infants up to 4 months of age.	Significantly different SM and fatty acids patterns were observed between groups. However, the profiles did not directly represent the dietary fatty acid pattern.	None: No differences between groups in weight gain or adverse events was observed.	[162]
WPC	IF, SF with MFGM-10 or with probiotic (*L. paracasei* ssp. *paracasei* strain F19) along with a BF reference group (*n* = 200 per group) fed for 4 months and followed to 12 months.	MFGM formula group did not have significantly more diarrhea, fever, days with fever, clinic visits, or URI episodes than the other formula groups or the BF infants.	None: Both experimental formulas were well tolerated and supported normal growth. Adverse event rates were highest in the control formula group with significantly more fever episodes and days with fever than the BF reference group.	[9]
WPC	IF provided 2 weeks through 12 months with either SF (*n* = 208) or MFGM-10 at 5 g/L with Lactoferrin (0.6 g/L (*n* = 198). Children were followed to 18 months of age.	MFGM+Lf formula group had higher cognitive, language, and motor Bayley-III scores at 12 months, better sustained attention at 12 months, and higher scores on some elements of language performance at 18 months, and were not inferior to the control group in any neurodevelopmental measure.	None: No significant differences between groups in growth, intolerance, fussiness, or stool characteristics were observed. Adverse event analysis demonstrated no differences between groups in antibiotic use and significantly lower incidences of GI and respiratory adverse events (including diarrhea, URI, and cough) for the MFGM + Lf formula group vs. the control group.	[163]
WPC + other bioactive ingredients	IF added MFGM-10, GNGL, LC-PUFA’s, sialic acid, and synbiotics (*n* = 85) compared to a control formula (*n* = 85) and BF reference group (*n* = 50). Term infants fed from <2 months of age until 18 months.	There were no differences in growth or neurodevelopment between formula groups; however, visual function was found to be more similar to the BF reference group.	None: No difference in weight/length gain between formula groups was observed.	[164]
